# Reduced gastrointestinal iron uptake in pulmonary arterial hypertension: a prospective cross-sectional study

**DOI:** 10.1186/s12931-026-03592-8

**Published:** 2026-03-02

**Authors:** Luisa Repsold, Satenik Harutyunova, Ekkehard Grünig, Nicola Benjamin, Panagiota Xanthouli, Benjamin Egenlauf, Andreea Florea, Memoona Shaukat, Richard Sparla, Christina Mertens, Martina U. Muckenthaler, Christina A. Eichstaedt

**Affiliations:** 1 Center for Pulmonary Hypertension, Thoraxklinik Heidelberg gGmbH at Heidelberg University Hospital, Röntgenstrasse 1, Heidelberg, 69126 Germany; 2 Translational Lung Research Center Heidelberg (TLRC), German Center for Lung Research (DZL), Heidelberg, Germany; 3 Department of Pneumology and Critical Care Medicine, Thoraxklinik Heidelberg gGmbH at Heidelberg University Hospital, Heidelberg, Germany; 4 Department of Internal Medicine V: Hematology, Oncology and Rheumatology, University Hospital Heidelberg, Heidelberg, Germany; 5 Centre for Translational Biomedical Iron Research, Department of Pediatric Oncology, Hematology, Immunology and Pulmonology, Molecular Medicine Partnership Unit (MMPU), University Hospital Heidelberg, Heidelberg, Germany; 6 Laboratory for Molecular Genetic Diagnostics, Institute of Human Genetics, Heidelberg University, Heidelberg, Germany

**Keywords:** Iron deficiency, Iron supplementation, Transferrin saturation, General measures, Pulmonary hypertension

## Abstract

**Background:**

Iron deficiency (ID) is common in patients with pulmonary arterial hypertension (PAH) and is associated with worse clinical outcomes. The etiology of ID in PAH is poorly understood. The aim of this study was to systematically determine whether differences in oral iron absorption exist between PAH patients with and without chronic ID compared with healthy controls.

**Methods:**

This single-center prospective, cross-sectional cohort study enrolled 45 subjects: 15 PAH patients with chronic ID, 15 PAH patients without ID and 15 healthy age and sex matched controls. Chronic ID was defined by either recorded ID anemia or clinical indication for i.v. iron supplementation in the past 3 years. Plasma iron levels and transferrin saturation (TSAT) were measured before and after a standardized oral iron absorption test with 200 mg ferrous iron. Additional iron and inflammation laboratory parameters were determined. Hepcidin and erythroferrone levels were measured using enzyme-linked immunosorbent assay, and tumor necrosis factor alpha and ferroportin expression were determined by quantitative polymerase chain reaction.

**Results:**

Both PAH groups showed a similar increase of plasma iron and TSAT after 3 h. The increase in plasma iron and TSAT was significantly lower in both PAH groups (with chronic ID: 71.5 (IQR 44.1–188.8) µg/dl; 22 (IQR 14–49) %; without ID: 86.0 (IQR 27.9–105.6) µg/dl; 26 (IQR 11–42) %) compared to healthy controls (154.1 (IQR 129.0–181.5) µg/dl; 45 (IQR 34–54) %, *p* = 0.015 and *p* = 0.031, respectively).

**Conclusions:**

This study is the first to demonstrate a significantly reduced gastrointestinal iron uptake in PAH patients compared to healthy age and sex matched controls. Interestingly, PAH patients with chronic ID showed similar iron uptake levels as those without, suggesting that factors other than iron stores, such as chronic inflammation, may impair iron absorption in this patient population.

## Introduction

Pulmonary arterial hypertension (PAH) is characterized by an increased pulmonary vascular resistance, right ventricular hypertrophy and ultimately right heart failure caused by a vascular remodeling of the lung [1].

Iron deficiency (ID) is common in PAH patients and is often recurrent despite regular intravenous (i.v.) iron supplementations [2]. Thus, not only acute ID or ID anemia, which is defined by a hemoglobin value < 12 g/dL or < 13 g/dL in women and in men, respectively, but also chronic ID affects PAH patients. The exact prevalence of ID in PAH patients varies widely between 23 and 68% depending on which definition is applied [3]. In the current European Society of Cardiology / European Respiratory Society (ESC/ERS) guidelines for the diagnosis and treatment of PH, ID is defined as ferritin < 100 µg/L or ferritin 100–299 µg/L and additionally a transferrin saturation (TSAT) < 20% [1]. However, other definitions are also in use. Some apply the same parameters, such as ferritin < 30 µg/L and TSAT < 16%, ferritin < 100 µg/L and TSAT < 20%, while others relied on parameters such as the soluble transferrin receptor (sTfR) index > 3.2 with a C-reactive protein < 5 mg/L [3, 4]. The latter definition can improve diagnostic accuracy, particularly in cases with inflammation-related alterations in ferritin levels [3] and to identify patients with more severely impaired hemodynamics [4].

Anemia and ID independent of anemia were reported to worsen the outcome of PAH patients including exercise capacity and mortality [5]. ID itself may even increase pulmonary pressures and drive pulmonary arterial remodeling [5–7]. More than 2% of hypochromic erythrocytes, reflecting impaired iron availability over the preceding three months, was found to be prognostically significant for a shorter survival in patients with PAH at baseline and follow-up [8, 9].

Iron substitution in anemic and iron deficient PAH patients is part of the general recommended measures for PAH patients [1]. A correction of iron status is recommended in the presence of ID anemia (Class I, Level C), while iron repletion may be considered in those with ID even in the absence of anemia (Class IIb, Level C) [1]. Studies showed that oral [10] or i.v [11, 12]. iron supplementation in iron deficient PAH patients led to an improvement in exercise capacity and endurance. A larger randomized controlled trial testing i.v. iron supplementation vs. placebo could, however, not confirm these results [13]. Thus, it is essential to characterize those patients who benefit from iron supplementation and understand the cause of ID. Recently, we could show that ID in idiopathic PAH (IPAH) patients and heritable PAH (HPAH) patients was not due to inappropriately elevated levels of the iron hormone hepcidin [2] as it has been previously suggested [5]. Instead, we demonstrated iron regulation to act as physiologically expected in PAH patients including patients with a pathogenic variant in the *BMPR2* gene, an upstream regulator of hepcidin [2].

Another explanation for ID in PAH patients could be an impaired gastrointestinal iron absorption [14]. However, this hypothesis has not yet been tested in a systematic and controlled manner.

Therefore, the aim of this study was to evaluate with a standardized protocol, whether oral iron absorption was reduced in PAH patients with chronic ID compared to those without chronic ID and age- and sex-matched healthy controls.

## Methods

### Study design

The cohort study was of explorative, cross-sectional, single-center nature carried out at the Center for Pulmonary Hypertension at the Thoraxklinik of Heidelberg University Hospital. The study protocol was approved by the ethics committee of the Medical Faculty of Heidelberg University (reference number: S-533/2023). Written informed consent was obtained from all participants. The authors confirm that the study was conducted in accordance with the Declaration of Helsinki in its latest version.

### Study population

of the final enrolement included 45 participants, split into 15 PAH patients with chronic ID, 15 PAH patients without prior ID, and 15 age- and sex-matched healthy controls. Chronic ID was defined as either a documented diagnosis of ID anemia or clinically indicated i.v. iron supplementation at least twice within the past three years. Clinical indication for iron infusion was an absolute iron deficiency with ferritin < 40 µg/l with or without anemia. TSAT was considered to rule out false negative laboratory results with too high ferritin values due to other reasons (e.g. chronic inflammation). Inclusion criteria for PAH patients were adult patients with a diagnosis confirmed by right heart catherization belonging with either an idiopathic, heritable, drug or toxin-induced or associated form of PAH with simple congenital heart disease or PAH with comorbidities not affecting iron metabolism. The hemodynamic criteria at diagnosis measured by right heart catheterization included a mean pulmonary arterial pressure > 20 mmHg, pulmonary arterial wedge pressure ≤ 15 mmHg and a pulmonary vascular resistance > 2 Wood units. Patients were on stable targeted PAH therapy for a minimum of two months before enrolment. Healthy controls were ≥ 18 years old and free from relevant cardiovascular or pulmonary diseases. Exclusion criteria for the full cohort were pregnancy or lactation, malignancies, severe kidney disease (glomerular filtration rate < 30 ml/min/1.73 m^2^), significant liver injury, iron supplementation within the last two months, transferrin saturation > 90%, acute infection or diarrhea.

### Study procedures

**Fig. 1:**
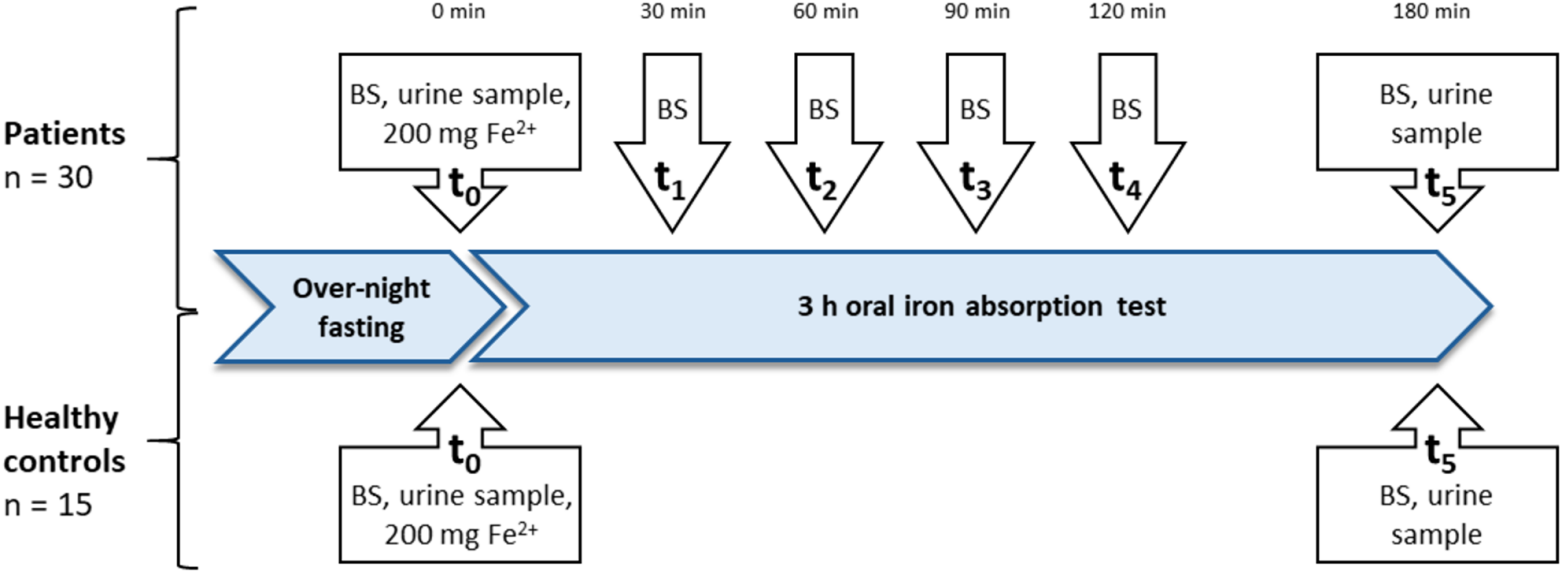
Study timeline. At baseline participants received 200 mg ferrous iron. Blood samples were taken at six time points in patients and two time points in healthy controls. All participants provided a urine samples before and after the test. BS = blood sample; Fe^2+^ = ferrous iron

All participants underwent a standardized oral iron absorption test starting approximately 9 am after fasting over night [15]. At baseline (t_0_) blood and urine were sampled. All participants received 567.7 mg oral ferrous glycine sulfate equivalent to 200 mg ferrous iron (Fig. 1). In patients, follow-up blood samples were collected every 30 to 60 min (t_1_-t_4_) totaling to an additional 20 ml of blood withdrawal each. After 3 h a second urine and the final blood sample was taken (t_5_). Urine was tested for its iron concentration via atomic absorption spectrometry. In healthy controls, only t_0_ and t_5_ samples were collected. In addition to the routine laboratory tests, the inflammatory markers interleukin 6 and leucocytes and the iron-related markers hypochromic erythrocytes, sTfR, erythropoietin and myoglobin were analyzed.

During the patients’ visit, clinical data were collected, including a detailed medical history, World Health Organization functional class (WHO-FC) and medication intake were documented. A comprehensive clinical assessment was conducted, including physical examination, echocardiography, electrocardiography (ECG), lung function testing including measurement of diffusion capacity for carbon monoxide (DLCO), as well as an arterial blood gas analysis. Functional capacity was assessed by the 6-minute walking test (6MWD). In addition, all study participants were asked to complete a questionnaire on dietary habits, history of ID or anemia, anticoagulant use, gastrointestinal issues, menstruation severity, and physical activity levels.

### Protein expression analyses

Serum samples for expression analyses were frozen at -80 °C. Enzyme-linked immunosorbent assays (ELISAs) were employed to determine the concentrations of the iron regulatory hormone and uptake inhibitor hepcidin as well as its regulator erythroferrone. For hepcidin the Hepcidin 25 (bioactive) HS ELISA Kit (DRG Instruments, Germany; protocol version 5.0) was used. Erythroferrone levels were assessed using the Intrinsic Erythroferrone ELISA Kit (Intrinsic LifeSciences, USA; protocol version 1.6). In both assays absorbance was recorded for technical duplicates at 450 nm and for hepcidin an additional background measurement was taken at 620 nm both using a SpectraMax M2e microplate reader (Molecular Devices, USA). Final levels were computed using the SoftMax Pro software (Molecular Devices, USA; version 6.5.1).

### RNA expression analyses

Peripheral blood was drawn into vacuum PAXgene Blood RNA tubes (BD, Germany) and frozen at -30 °C until further processing. Manual or semi-automated RNA extraction was carried out with the PAXgene Blood RNA Kit (PreAnalytiX, Switzerland) according to the company’s protocol (version 3). Resulting concentrations were measured employing NanoDrop ND-1000 spectrophotometer (Peglab, Germany). RNA were frozen at -80 °C. Complementary DNA was produced with the Transcriptor First Strand cDNA Synthesis Kit (Roche, Switzerland; protocol version 09) and also stored at -80 °C. Quantitative polymerase chain reaction was carried out on a StepOnePlus Real-Time PCR System (Thermofisher Scientific, USA) using SYBR Green Master Mix (Thermofisher Scientific, USA) and gene-specific primers (Sigma-Aldrich, USA) for Ferroportin and tumor necrosis factor alpha (TNF α). The delta cycle threshold (∆CT) method was used to obtain gene expression relative to the housekeeping gene glyceraldehyde-3-phosphate dehydrogenase (*GAPDH*) using the StepOne Software (Thermofisher Scientific, USA; Version 2.3.). All reactions were performed as technical duplicates.

### Statistical analyses

Baseline characteristics were summarized with descriptive statistics, clinical and laboratory data, with measures including mean, median, interquartile range, minimum, maximum, and 95% confidence intervals. For categorical variables, frequency tables were provided. Change in plasma iron levels from baseline to 3 h after iron supplementation was the primary endpoint of this study. Comparisons between patients with chronic ID and patients without ID and healthy controls were performed by non-parametric Kruskal-Wallis H test. For three group comparisons and for non-parametric-intergroup comparisons at a two-sided significance level of α = 0.05 the Wilcoxon-Mann-Whitney-U test was used. Chi-square test was employed for categorical variables. Secondary analyses included group comparisons of laboratory and clinical parameters, as well as correlation analyses with plasma iron levels. Mutlivariable regression analysis with stepwise forward selection was used to identify significant inflammatory markers as determinants of iron uptake in PAH patients. Due to the exploratory nature of the study, correction for multiple testing was not applied. Statistical calculations were performed with SPSS (version 29.0, IBM Corp., Somers, New York, USA).

## Results

### Baseline characteristics

Overall, 45 individuals were enrolled in the study between October 2023 and April 2024, including 30 patients with PAH, 15 with chronic ID and 15 without ID, and 15 age- and sex-matched healthy participants. A standardized oral iron absorption test was conducted for all participants as well as an extended assessment of iron metabolism in addition to routine diagnostic evaluations.

In Table 1 clinical baseline characteristics of both patient groups are shown. PAH patients with chronic ID were more often female (93.3% vs. 60.0%) and were slightly younger than PAH patients without ID (median age 50 (interquartile range (IQR) 36–64) vs. 59 (IQR 45–77) years), though differences were not statistically significant. PAH subtypes, WHO-FC, and treatment strategies were similar across groups. Echocardiographic, hemodynamic, and functional parameters, including 6MWD and DLCO, also showed no significant differences. Only peripheral oxygen saturation was significantly lower in patients with chronic ID (*p* = 0.006). The baseline characteristics of PAH patients with and without chronic ID were comparable. The healthy control group consisted of 15 individuals (9 females, 6 males) with a median age of 46 (IQR 38–56) years. None of the participants were vegetarians or vegans (Table 2). Since 23 out of 32 women in the study were post-menopausal, the recorded numbers for menstruation severity from light to very strong were too low to analyze.

**Table 1:** Clinical data of PAH patients with and without chronic ID

Characteristics	PAH patients with chronic ID (*n* = 15)		PAH patients without ID (*n* = 15)		
	Median (IQR) or *n* [%]	*n*	Median (IQR) or *n* [%]	*n*	*p*-value
Female, sex no [%]	14 [93.3%]	15	9 [60.0%]	15	0.080
Body mass index [kg/m^2^]	24.4 (21.5–33.1)	15	25.3 (22.8–31.6)	15	0.660
Systolic blood pressure [mmHg]	114 (105–121)	15	110 (101–119)	15	0.999
Diastolic blood pressure [mmHg]	70.0 (68.0–77.0)	15	71.0 (62.0–82.0)	15	0.660
Age [years]	50.0 (36–64)	15	59.0 (45–77)	15	0.375
Type of PAH		15		15	0.967
IPAH	8 [53.3%]		9 [60.0%]		
HPAH	3 [20.0%]		2 [13.3%]		
CHD-APAH	1 [6.7%]		1 [6.7%]		
DPAH	0 [0.0%]		0 [0.0%]		
PAH with comorbidities	3 [20.0%]		3 [20.0%]		
WHO-FC, no [%]		15		15	0.256
II	11 [73.3%]		8 [53.3%]		
III	4 [26.7%]		7 [46.7%]		
Targeted PAH therapy		15		15	
Endothelin receptor antagonist	13 [86.7%]		9 [60.0%]		0.215
PDE5 inhibitor/sGC stimulator	14 [93.3%]		14 [93.3%]		1.000
Prostacyclin	5 [33.3%]		3 [20.0%]		0.682
Calcium channel blocker	1 [6.7%]		1 [6.7%]		1.000
Mono therapy	1 [6.7%]		6 [40.0%]		0.091
Double therapy	9 [60.0%]		6 [40.0%]		
Triple therapy	5 [33.3%]		2 [13.3%]		
Quadruple therapy	0 [0.0%]		1 [6.7%]		
Echocardiography					
sPAP [mmHg]	41.0 (30.0–50.0)	14	40.0 (31.0–71.0)	12	0.973
Right atrial area [cm^2^]	16.0 (12.0–21.0)	15	18.0 (14.0–30.0)	15	0.375
Right ventricular area [cm^2^]	18.0 (13.0–19.0)	15	19.0 (13.0–28.0)	15	0.660
TAPSE [mm]	24.0 (20.0–28.0)	15	21.0 (18.0–24.0)	15	0.660
Most recent right heart catheterization					
mPAP [mmHg]	39.0 (34.0–45.0)	15	35.0 (31.0–56.0)	15	0.660
PAWP [mmHg]	10.0 (6.0–12.0)	15	10.0 (7.0–12.0)	15	0.925
PVR [WU]	5.67 (4.60–7.73)	15	6.69 (3.17–10.71)	14	0.660
Cardiac output [l/min]	4.70 (4.00-5.35)	15	5.13 (3.50–6.50)	15	0.299
Cardiac index [l/min/m^2^]	2.80 (2.44-3.00)	15	2.60 (1.73–3.05)	14	0.553
6-minute walking test					
Distance [m]	486 (380–510)	13	420 (377–489)	13	0.570
Lung function testing					
DLCO SB [%]	68.0 (58.0–76.0)	15	51.0 (33.0–69.0)	15	0.076
VC max [%]	88.0 (74.0-102.0)	15	82.0 (78.7–94.0)	14	0.682
FEV1 [%]	83.0 (69.0–95.0)	15	75.5 (66.9–88.0)	14	0.208
pO_2_ [mmHg]	70.0 (63.0–80.0)	15	65.0 (59.0–74.0)	15	0.660
SpO_2_ at rest [%]	96.0 (94.0–98.0)	15	98.0 (97.0–99.0)	15	0.006*

*CHD-APAH* Pulmonary arterial hypertension associated with congenital heart disease, *DLCO SB* Diffusion capacity of the lung for carbon monoxide - single breath, *DPAH* Drug and toxin associated pulmonary arterial hypertension, *FEV1* Forced expiratory volume in 1 s, *HPAH* Heritable pulmonary arterial hypertension, *ID* Iron deficiency, *IPAH* Idiopathic pulmonary arterial hypertension, *IQR* Interquartile range, *mPAP* Mean pulmonary arterial pressure, *PAH* Pulmonary arterial hypertension, *PAWP* Pulmonary arterial wedge pressure, *PDE5* Phosphodiesterase type 5, *pO*_2_ Partial pressure of oxygen, *PVR* Pulmonary vascular resistance, *sGC* Soluble guanylate cyclase, *sPAP* Systolic pulmonary arterial pressure, *SpO*_2_ Peripheral oxygen saturation, *TAPSE* Tricuspid annular plane systolic excursion, *VC max* Maximal vital capacity, *WHO-FC* World Health Organization functional class

*p < 0.05

Baseline laboratory parameters revealed significant differences in iron metabolism between the three groups (Table 2). Plasma iron levels at baseline significantly differed between groups (*p* = 0.007) and were the lowest in PAH patients without ID (60.9 µg/dl), compared to those with ID (72.0 µg/dl) and healthy controls (101.6 µg/dl). Mean corpuscular volume was the highest in patients with ID (*p* = 0.025), albeit still within the normal range. The percentage of hypochromic erythrocytes was slightly but significantly increased in patients without ID compared to both other groups albeit still being within the normal range (*p* = 0.016). Soluble transferrin receptor (sTfR) concentrations were increased in PAH patients without ID (*p* = 0.026), while erythropoietin (EPO) levels were highest in PAH patients with chronic ID (*p* = 0.028).

**Table 2:** Baseline (t_0_) laboratory and questionnaire parameters in study participants

Parameter	PAH patients with chronic ID (*n* = 15)		PAH patients without ID (*n* = 15)		Healthy controls (*n* = 15)		
	Median (IQR) or *n* [%]	*n*	Median (IQR) or *n* [%]	*n*	Median (IQR) or *n* [%]	*n*	*p*-value
Iron parameters							
Plasma iron [µg/dl]	72.0 (65.9-113.4)	15	60.9 (40.2–82.7)	15	101.6 (71.5–115.0)	15	0.007*
TSAT [%]	25 (20–38)	15	20 (12–31)	15	32 (23–36)	15	0.057
Transferrin [g/l]	2.10 (2.03–2.26)	15	2.03 (1.86–2.35)	15	2.22 (2.09–2.55)	15	0.246
Erythrocytes [/pl]	4.6 (4.4–4.9)	15	4.7 (4.4-5.0)	15	4.8 (4.5–5.3)	15	0.653
Hemoglobin [g/dl]	14.2 (13.1–15.7)	15	14.1 (13.0-15.2)	15	14.6 (13.2–15.4)	15	0.743
Hematocrit [l/l]	0.41 (0.40–0.45)	15	0.42 (0.37–0.45)	15	0.43 (0.39–0.44)	15	0.903
MCV [fl]	91 (88–92)	15	87 (84–90)	15	87 (85–89)	15	0.025*
MCH [pg]	34 (33–35)	15	30 (29–30)	15	30 (29–31)	15	0.054
MCHC [g/dl]	34 (33–35)	15	34 (33–34)	15	34 (34–35)	15	0.417
Rbdcw [%]	13.2 (12.7–13.6)	15	13.9 (13.2–14.6)	15	12.6 (12.2–12.6)	15	< 0.001*
Hypochromic erythrocytes [%]	0.1 (0.1–0.2)	15	0.2 (0.1–0.3)	15	0.1 (0.1–0.2)	15	0.016*
Ferritin [µg/l]	72 (24–128)	15	86 (63–124)	15	51 (35–67)	15	0.143
sTfR [mg/l]	2.8 (2.6–3.1)	15	3.2 (2.6–4.5)	15	2.6 (2.3–2.8)	15	0.026*
Ferritin index	1.38 (1.27-2.00)	15	1.71 (1.36–2.38)	15	1.44 (1.22–1.66)	15	0.297
EPO [mU/ml]	11.9 (8.3–16.9)	15	8.6 (6.4–17.1)	15	6.9 (5.8–10.9)	15	0.028*
Myoglobin[µg/l]	32 (23–44)	15	49 (40–60)	15	41 (31–49)	15	0.023*
Hepcidin [ng/ml]	11.0 (7.2–23.3)	15	15.5 (12.6–16.2)	15	9.8 (6.5–18.1)	15	0.385
Erythroferrone [ng/ml]	0.63 (0.33–1.80)	15	0.49 (0.29–1.70)	15	0.28 (0.11–1.18)	12	0.319
Ferroportin mRNA expression	0.19 (0.17–0.20)	15	0.23 (0.14–0.25)	15	0.13 (0.12–0.21)	14	0.229
Urinary iron [µmol/l]	0.10 (0.07–0.43)	15	0.13 (0.07–0.27)	15	0.22 (0.10–0.34)	15	0.546
Inflammatory markers							
CRP [mg/l]	2.0 (2.0-2.1)	15	8.4 (4.0–9.0)	15	2.0 (2.0–2.0)	15	< 0.001*
Leukocytes [/nl]	6.66 (5.58–7.46)	15	6.32 (5.36–8.28)	15	5.99 (4.75–7.53)	15	0.641
Neutrophils [/nl]	4.26 (3.11–4.93)	15	3.28 (2.66–5.83)	15	3.08 (2.32–4.98)	15	0.200
Lymphocytes [/nl]	1.53 (1.24–1.93)	15	1.64 (1.11–2.05)	15	1.78 (1.56–2.41)	15	0.217
Monocytes [/nl]	0.45 (0.39–0.55)	15	0.47 (0.38–0.51)	15	0.51 (0.36–0.62)	15	0.841
Eosinophils [/nl]	0.13 (0.04–0.24)	15	0.18 (0.12–0.21)	15	0.15 (0.12–0.34)	15	0.378
Basophiles [/nl]	0.05 (0.04–0.07)	15	0.05 (0.03–0.07)	15	0.04 (0.04–0.06)	15	0.936
IL6 [pg/ml]	2.0 (2.0-3.3)	15	2.6 (2.0–5.0)	15	2.0 (2.0–2.0)	15	0.022*
TNF α mRNA expression	0.02 (0.01–0.02)	15	0.02 (0.01–0.02)	15	0.02 (0.02–0.02)	14	0.683
Other parameters							
GFR [ml/min/1.73 m^2^]	97 (60–104)	15	86 (60–106)	15	100 (82–112)	15	0.356
Creatinine [mg/dl]	0.74 (0.67–0.91)	15	0.86 (0.71–1.08)	15	0.81 (0.70–0.88)	15	0.434
NT-proBNP [ng/l]	306 (69–987)	15	547 (222–2336)	15	61 (35–67)	15	< 0.001*
Questionnaire							
Diet		15		15		15	0.054
Pescetarian	0		0		1 [7%]		
No meat restrictions	15 [100%]		15 [100%]		14 [93%]		
Co-medication		15		15		15	
Anticoagulant use	10 [67%]		13 [87%]		0		< 0.001*
Proton pump inhibitors	4 [27%]		7 [47%]		0		0.012*
Gastrointestinal problems	5 [33%]	15	3 [20%]	15	1 [7%]	15	0.167
Physical activity	12 [80%]	15	8 [53%]	15	5 [33%]	15	0.301

*CRP* C-reactive protein, *EPO* Erythropoietin, *GFR* Glomerular filtration rate, *ID* Iron deficiency, *IL6* Interleukin 6, *IQR* Interquartile range, *MCH* Mean corpuscular hemoglobin, *MCHC* Mean corpuscular hemoglobin concentration, *MCV* Mean corpuscular volume, *NT-proBNP* N-terminal pro brain natriuretic peptide, *Rbdcw* Red blood cell distribution width, *TNF α* Tumor necrosis factor alpha, *TSAT* Transferrin saturation, *sTfR* Soluble transferrin receptor

*p < 0.05

Three patients (7%) showed acute anemia at baseline with a hemoglobin value below 12 g/dl in female or 13 g/dl in male participants. However, considering the new very broad ESC/ERS PH guidelines definition for ID 78% of all participants would have been considered iron deficient (ferritin < 100 µg/L or ferritin 100–299 µg/L and a transferrin saturation (TSAT) < 20% [1].

The inflammatory markers C-reactive protein (CRP) and interleukin 6 (IL6) were mildly but significantly increased in PAH patients without ID (CRP: *p* < 0.001; IL6: *p* = 0.022, Table 2). Due to right heart insufficiency, N-terminal pro brain natriuretic peptide (NT-proBNP) levels were strongly augmented in patients compared to controls (*p* < 0.001).

Four patients in the chronic ID group (27%) and seven patients in the non-ID group (47%) were taking proton pump inhibitors, whereas none of the healthy controls reported their use (*p* = 0.003).

### Standardized iron absorption test

**Fig. 2:**
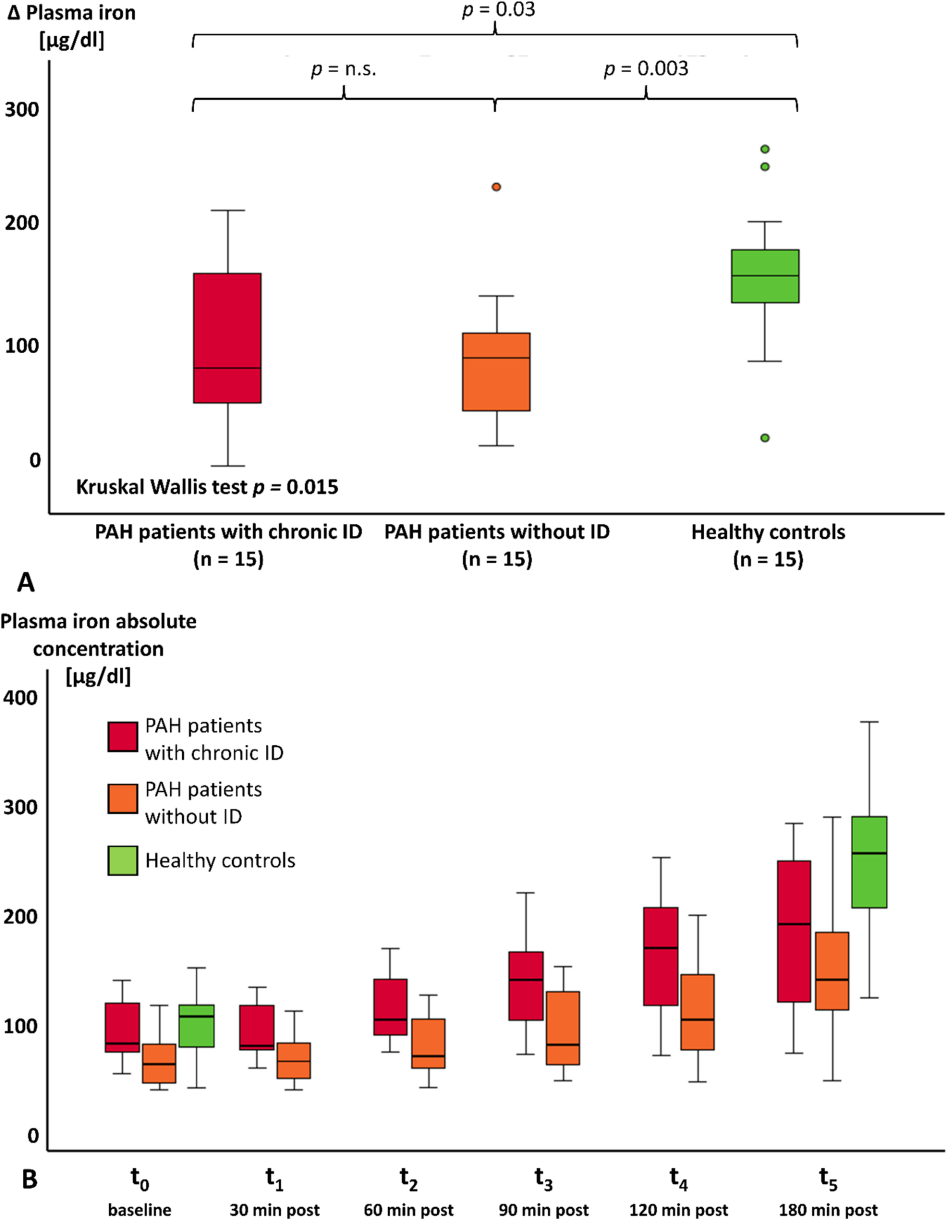
Difference and increase of iron levels over 3 h in PAH patients and healthy controls. **A** Both patient groups showed a significantly lower increase in plasma iron compared to healthy controls. **B** Both patient groups had a similar increase in plasma iron over time. PAH = pulmonary arterial hypertension

Three hours following the oral uptake of 200 mg ferrous iron blood iron levels increased in both patient groups and healthy controls (Fig. 2A). Plasma iron levels rose similarly in both PAH groups, with increases of 71.5 (IQR 44.1–188.8) µg/dL in patients with chronic ID and 86.0 (IQR 27.9–105.6) µg/dL in those without ID. However, healthy controls exhibited a significantly greater increase in plasma iron (154.1 (IQR 129.0–181.5) µg/dL) compared to both patient groups with chronic ID (*p* = 0.03) and without ID (*p* = 0.003) despite even higher starting levels. Fig. 2B illustrates the plasma iron concentrations over six time points, showing that PAH patients without ID displayed a slightly lower but comparable increase in plasma iron relative to PAH patients with chronic ID.

**Fig. 3:**
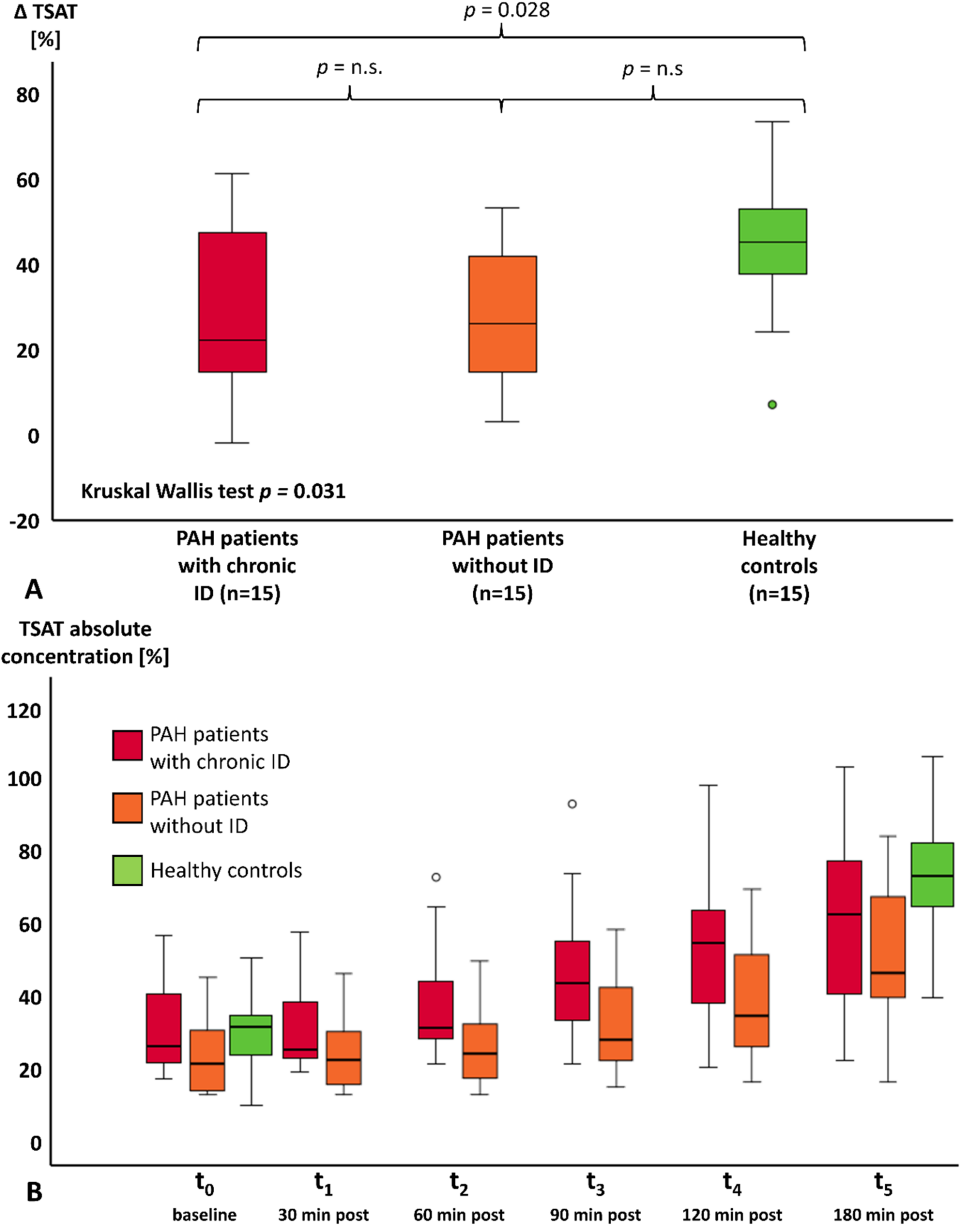
Difference and increase of transferrin saturation over 3 h in PAH patients and healthy controls. **A** Patients showed a significantly lower increase in TSAT compared to healthy controls. **B** Both patient groups showed a similar increase in TSAT over the time of 3 h. PAH = pulmonary arterial hypertension; TSAT = transferrin saturation; t_0_ = baseline, t_1_ = 30 min, t_2_ = 60 min, t_3_ = 90 min t_4_ = 120 min, t_5_ = 180 min

Similar to plasma iron, transferrin saturation (TSAT) also increased in all groups following iron administration (Fig. 3A). Again, the rise was similar in both PAH groups (PAH with chronic ID: 22% (IQR 14–49%); PAH without ID: 26% (IQR 11–42%)), but significantly enhanced in healthy controls (45% (IQR 34–54%); *p* = 0.031). PAH patients without ID showed a slightly lower but comparable TSAT response to those with ID (Fig. 3B).

No significant differences in urinary iron concentrations could be detected in any group after 3 h.

Results did not significantly differ between patients with or without proton pump inhibitor (PPI) intake for iron uptake (no PPI use median 77.63, IQR 46.35–107.78 µg/dl vs. PPI use median 86.00 IQR 24.57-136.27 µg/dl) and for transferrin saturation (no PPI use median 23.00 IQR 14.00–42.00 vs. PPI use median 25.00 IQR 11.00–53.00).

### Relationship of iron status with selected clinical and inflammation parameters in PAH patients

At baseline (t_0_), no significant correlations for iron parameters (plasma iron, TSAT, ferritin, and hepcidin) and inflammatory markers (CRP, leukocytes, IL6, and TNF α) could be detected across patient groups. As physiologically expected, higher ferritin strongly correlated with higher hepcidin (*p* < 0.001; Table 3), while hepcidin showed a negative correlation with erythroferrone (*p* = 0.047) and EPO (*p* = 0.020). In concert, a positive correlation was identified between EPO and erythroferrone (*p* = 0.043). Additionally, ferroportin mRNA levels correlated positively with leukocyte (*p* = 0.014) and neutrophil counts (*p* = 0.005) at t_0_. After three hours (t_5_), ferroportin levels correlated with both plasma iron and TSAT (both *p* = 0.009, Table 3).

**Table 3:** Correlations of iron and immune-related parameters in PAH patients

Variable A	Variable B	*r*	*p*-value
Hepcidin t_0_	Plasma iron t_0_	0.088	0.644
Hepcidin t_0_	TSAT t_0_	0.247	0.189
Hepcidin t_0_	Ferritin t_0_	0.810	< 0.001*
Hepcidin t_0_	Erythroferrone t_0_	-0.366	0.047*
Hepcidin t_0_	EPO t_0_	-0.424	0.020*
Hepcidin t_0_	Leukocytes t_0_	-0.086	0.651
Hepcidin t_0_	Neutrophils t_0_	-0.072	0.707
EPO t_0_	Erythroferrone t_0_	0.327	0.043*
Ferroportin t_0_	Plasma iron t_0_	0.224	0.234
Ferroportin t_0_	TSAT t_0_	0.283	0.130
Ferroportin t_0_	Ferritin t_0_	-0.061	0.751
Ferroportin t_0_	Erythroferrone t_0_	0.016	0.934
Ferroportin t_0_	EPO t_0_	0.084	0.659
Ferroportin t_0_	Leukocytes t_0_	0.444	0.014*
Ferroportin t_0_	Neutrophils t_0_	0.498	0.005*
Ferroportin t_5_	Plasma iron t_5_	0.476	0.009*
Ferroportin t_5_	TSAT t_5_	0.475	0.009*
Ferroportin t_5_	Ferritin t_5_	0.057	0.770
Ferroportin t_5_	Erythroferrone t_5_	-0.093	0.633
Ferroportin t_5_	EPO t_5_	0.015	0.939
Ferroportin t_5_	Leukocytes t_5_	0.342	0.069
Ferroportin t_5_	Neutrophils t_5_	0.367	0.050

*EPO* Erythropoietin, *TSAT* Transferrin saturation, *t*_0_ baseline, *t*_5_ 180 min

*p < 0.05

Correlations between iron status and selected clinical parameters in PAH patients are shown in Table 4. Considering the increase in plasma iron over 3 h, greater changes correlated with a higher cardiac output in patients (*r* = 0.368, *p* = 0.045). In contrast, TSAT changes over 3 h revealed no correlations with any cardiopulmonary parameters in patients.

**Table 4:** Correlations of iron status and selected clinical parameters in PAH patients

Variable A	Variable B	*r*	*p*-value
Plasma iron t_5_-t_0_	CI (most recent RHC)	0.368	0.050
Plasma iron t_5_-t_0_	CO (most recent RHC)	0.368	0.045*
Plasma iron t_5_-t_0_	RV function impairment	0.137	0.479
Plasma iron t_5_-t_0_	SpO_2_	0.135	0.477
Plasma iron t_5_-t_0_	6MWD	0.158	0.440
Plasma iron t_5_-t_0_	WHO-FC	-0.260	0.166
Ferritin t_0_	CI (most recent RHC)	-0.370	0.148
Ferritin t_0_	CO (most recent RHC)	-0.292	0.117
Ferritin t_0_	RV function impairment	-0.426	0.021*
Ferritin t_0_	SpO_2_	0.369	0.045*
Ferritin t_0_	6MWD	-0.240	0.238
Ferritin t_0_	WHO-FC	-0.120	0.528
Hepcidin t_0_	CI (most recent RHC)	-0.331	0.019*
Hepcidin t_0_	CO (most recent RHC)	-0.300	0.108
Hepcidin t_0_	RV function impairment	-0.246	0.198
Hepcidin t_0_	SpO_2_	0.204	0.280
Hepcidin t_0_	6MWD	-0.260	0.200
Hepcidin t_0_	WHO-FC	-0.060	0.753
sTfR t_0_	CI (most recent RHC)	0.009	0.961
sTfR t_0_	CO (most recent RHC)	0.128	0.499
sTfR t_0_	RV function impairment	-0.284	0.136
sTfR t_0_	SpO_2_	-0.020	0.917
sTfR t_0_	6MWD	-0.080	0.696
sTfR t_0_	WHO-FC	0.044	0.817

*CI* Cardiac index, *CO* Cardiac output, *RHC* Right heart catheterization, *RV* Right ventricular, *SpO2* Peripheral oxygen saturation, *sTfR* Soluble transferrin receptor, *t*0 = baseline, *t*5 180 min, *WHO-FC* World Health Organization functional class, *6MWD* Six-minute-walking-distance

*p < 0.05

Higher ferritin levels at t_0_ correlated with a better right ventricular pump function (*r* = − 0.426, *p* = 0.021), a better peripheral oxygen saturation (SpO_2_) (*r* = 0.369, *p* = 0.045). but a lower cardiac index (*r* = -0.370, *p* = 0.048). Lower hepcidin levels correlated with both a better cardiac index at diagnosis and at its most recent measurement (*r* = -0.044, *p* = 0.019; *r* =-0.423, *p* = 0.022).

Within the three hour observation period, neutrophil counts increased similarly across all study groups (*p* = 0.073). However, following the iron absorption test (t_5_), a significant difference in neutrophil counts was observed specifically in PAH patients with chronic ID (*p* = 0.013), in contrast to both PAH patients without ID and healthy controls. For other recorded immune cells we found no evidence of a difference between the three groups after the iron absorption test (data not shown).

A stepwise forward multivariable regression analysis with IL-6, hepcidin and C-reactive protein identified hepcidin to be a significant determinant of iron increase (*R* = 0.525, B = -2.878, *p* = 0.003) during the iron absorption test in PAH patients, with higher values being correlated to lower increase if iron plasma levels.

## Discussion

We present the first study employing a systematic, standardized oral iron absorption test in PAH patients in comparison to age and sex matched healthy controls. We could reveal for the first time a drastically reduced increase in plasma iron and TSAT in PAH patients vs. controls. This impaired gastrointestinal iron uptake could offer an explanation for repeated ID in PAH patients.

### Significant impaired gastrointestinal iron uptake in PAH

Our data extend upon a previous study by others that also suggested an impaired iron uptake in PAH patients [16]. However, in this study the iron uptake was not assessed with a standardized iron absorption test and was not compared with healthy controls. Furthermore, in that study only 2/18 patients showed a significant increase in plasma iron after four weeks of iron intake, while TSAT remained below the normal range in 14/18 patients [16]. In contrast, in our study, we could demonstrate an increase of plasma iron and TSAT with the median of all three groups reaching or even exceeding the normal range of plasma iron and TSAT at the end of the 3 h observation. Regardless of the increase, there was a drastic and significant difference in the degree of iron uptake in the patients vs. healthy controls.

This impaired absorption response in PAH patients resembles the pattern observed in anemia of chronic disease (ACD) or functional ID rather than classic, acute ID anemia [17]. In a classic model of ID anemia, a strong increase in iron levels of more than 200 µg/dL was demonstrated already at a dose of 100 mg sodium ferrous citrate [17]. ACD is associated with chronic inflammation and characterized by impaired iron availability (hypoferremia) and utilization. In ACD, iron is sequestered within storage sites, specifically macrophages [6] and is not adequately accessible for erythropoiesis, despite normal or even increased total body iron stores [18].

In our study we tried to identify patients with chronic ID, yet a universally accepted diagnostic framework is currently lacking and would be very helpful for future studies. We could identify a few unexpected differences between the two groups in iron metabolism such as a slightly increased sTfR and red cell distribution width in patients without chronic ID. This may point towards a mild underlying acute ID also in this patient group.

### Further factors associated with iron deficiency in PAH

At present, no standardized definition for chronic ID in the context of PAH or other chronic diseases exists. However, our findings suggest there are mechanisms at play in PAH patients which inhibit iron absorption, contributing to a functional iron-restricted state despite preserved or only mildly reduced iron stores. To this end, inflammatory markers were only slightly elevated in PAH patients without ID. This borderline inflammation in some patients without ID may be a possible explanation for this group showing a non-significant but in trend lower absorption of iron.

We had also explored the option of active iron excretion via urine as it was shown for mice [19] but we could not detect any elevated iron levels in urine after 3 h in our participants. An investigation of iron excretion via stool and the involvement of the gut microbiome remains to be analysed in further studies. The frequent use of PPI in PAH patients vs. controls also offered no explanation for the diminished iron uptake supporting ACD as the most likely cause of disturbed iron uptake in the PAH patients.

While inflammatory markers were slightly elevated in patients without chronic ID, we could observe the highest EPO levels in patients with chronic ID. This may be explained by a compensatory feedback mechanism, in which the kidney increases EPO production in response to iron-restricted erythropoiesis in an attempt to stimulate red blood cell formation [20].

We also identified a significant increase in neutrophils across all groups after 3 h following the oral iron application with PAH patients with chronic ID showing the highest neutrophil levels. Neutrophils can temporarily adopt functions typically carried out by other cell types, such as macrophages, by acting as a buffer system that sequesters circulating iron [21]. While macrophages were not measured in our study it appears neutrophils acted as first responders to sudden increased iron levels. This mechanism has been described in the context of inflammation, where mostly macrophages limit iron availability to pathogens that rely on iron for growth and replication [22]. Supporting this, we observed positive correlations between plasma iron and TSAT with ferroportin levels at t_5_ compared to t_0_. Elevated ferroportin expression on neutrophil cells has been described, although the exact function of neutrophil-associated ferroportin remains unclear [23]. An alternative explanation could be, that iron homeostasis is critical for neutrophil development. Mature neutrophils can only be built, when intra cellular iron supply is granted [24], while differentiation of neutrophils is sensitive to rapid physiological changes in serum iron concentration [25]. Future studies with extended observation periods, functional assays and measurement of further immune cells may help elucidate the temporal dynamics of neutrophil and macrophage responses and their potential contribution to iron homeostasis in PAH.

### Iron deficiency correlated with more severe hemodynamic impairment

Our clinical correlation analyses confirmed that lower iron status was associated with a more severe clinical presentation, supporting previous findings of clinical deterioration in this context [5]. Consistent with earlier studies, we describe correlations between ID and reduced cardiac index [26], lower SpO₂ [5], and impaired right ventricular function [3, 27]. The observed correlation of lower hepcidin with a better cardiac index is supported by data from mouse models [28, 29]. In these a loss of cardiac hepcidin and reduction of the iron exporter ferroportin have been shown to cause iron dysregulation within cardiomyocytes, in particular the latter leading to progressive impairment of cardiomyocyte function and subsequently even cardiac failure [28, 29]. Thus, lower hepcidin could have enabled higher ferroportin within the heart allowing for better heart function in our cohort.

Correlations with other parameters previously linked to ID, such as 6MWD and WHO-FC [5], were not significant in our cohort. This could, however, also be due to different patient inclusion criteria as not acute but chronic ID was investigated in our study. Nonetheless, prior research has shown that ID is associated with worsening functional status and reduced survival [5, 9, 30].

Overall, the molecular correlations observed in this study are in line with established physiological mechanisms regulating iron homeostasis as previously described for IPAH and HPAH patients by our group [2]. As expected, erythroferrone was positively associated with EPO, regulating iron availability. EPO is produced in response to increased erythropoietic demand [31]. Erythroferrone, in turn, was inversely related to hepcidin levels, supporting its role as a hepcidin-suppressing factor [21].

### Limitations

Our study had a relatively small sample size. However, the study was powered to identify significant changes of plasma iron levels after 3 h which could be successfully detected. The study may however have been underpowered for additional outcome parameters and for subgroup analyses within the PAH cohort. Since the standardized iron absorption test has a duration of only 3 h our ability was limited to detect delayed or more subtle differences in iron absorption kinetics. Moreover, the study was laid-out as a single-center, cross-sectional, exploratory cohort study, which inherently limits causal inference and generalizability. Even though we focused on chronic ID by selecting individuals with a history of ID and / or requiring i.v. iron supplementation in the past three years, 78% of our cohort could have been defined as having an acute ID by using the very broad criteria of the ESC/ERS PH guidelines. Thus, even patients classified as not chronically iron deficient could have been considered acutely iron deficient applying these criteria. Hence, in future studies, more stringent criteria for chronic ID such as a certain number of i.v. supplementations due to repeatedly low iron parameters in a defined period of time may provide a clearer distinction between patients with and without chronic ID. Nevertheless, we decided to focus on chronic ID to better reflect the clinical relevance of long-standing iron depletion in PAH patients.

Although the study evaluates systemic iron handling, no gastrointestinal parameters (duodenal ferroportin, DMT1, inflammation, microbiome, stool iron) were assessed, which should be addressed in future projects.

## Conclusions

The standardized iron absorption test demonstrated a significant reduction in oral iron uptake in PAH patients compared to healthy controls. Whether i.v. iron supplementation in contrast to orally provided iron may be better suitable to increase iron levels in PAH patients remains to be investigated. Interestingly, PAH patients with chronic ID showed similar iron uptake levels as those without chronic ID, suggesting that factors other than iron stores, such as chronic inflammation, may impair iron absorption in this patient population. These results underline the complexity of iron metabolism in PAH and stress the need for additional studies to clarify the mechanisms involved and to develop tailored diagnostic and therapeutic approaches.

## Data Availability

All data generated or analysed during this study are included in this published article.

## Abbreviations

6MWD: Six-minute-walking-distance
ACD: Anemia of acute disease
CRP: C-reactive protein
∆CT: Delta cycle threshold
DLCO: Diffusion capacity for carbon monoxide
ECG: Electrocardiography
ELISA: Enzyme-linked immunosorbent assay
EPO: Erythropoietin
ESC/ERS: European Society of Cardiology / European Respiratory Society
HPAH: Heritable pulmonary arterial hypertension
ID: Iron deficiency
IL6: Interleukin 6
IQR: Interquartile range
IPAH: Idiopathic pulmonary arterial hypertension
i.v.: Intravenous
NT-proBNP: N-terminal pro brain natriuretic peptide
PAH: Pulmonary arterial hypertension
SpO_2_: Peripheral oxygen saturation
sTfR: Soluble transferrin receptor
TNFα: Tumor necrosis factor alpha
TSAT: Transferrin saturation
WHO-FC: World Health Organization functional class
